# Temporal and vertical variations of polycyclic aromatic hydrocarbon at low elevations in an industrial city of southern Taiwan

**DOI:** 10.1038/s41598-021-83155-7

**Published:** 2021-02-10

**Authors:** Wei-Hsiang Chen, Ming-Tsuen Hsieh, Jie-Yu You, Adnan Quadir, Chon-Lin Lee

**Affiliations:** 1 Institute of Environmental Engineering, National Sun Yat-Sen University, Kaohsiung, 804 Taiwan; 2 Aerosol Science Research Center, National Sun Yat-Sen University, Kaohsiung, 804 Taiwan; 3 Department of Public Health, Kaohsiung Medical University, Kaohsiung, Taiwan; 4 Department of Marine Environment and Engineering, National Sun Yat-Sen University, Kaohsiung, 804 Taiwan; 5 Department of Applied Chemistry, Providence University, Taichung, Taiwan

**Keywords:** Atmospheric chemistry, Environmental monitoring

## Abstract

Considered that human activities mostly occur below building heights, the objective of this study was to investigate the temporal variations of fine particular matter (PM_2.5_)-associated polycyclic aromatic hydrocarbons (PAHs) and benzo[a]pyrene-equivalent (BaP_eq_) concentrations at four different elevations (6.1, 12.4, 18.4, and 27.1 m) in Kaohsiung City, the largest industrial city of southern Taiwan. Temperature variation was critical for the PM_2.5_-associated PAH concentrations, which were dominated by benzo[g,h,i]perylene (0.27 ± 0.04 ng m^−3^ and 24.43% of the total concentration) and other high molecular weight (HMW) species. The PM_2.5_-associated BaP_eq_ was dominated by 5-ring PAH (36.09%). The PM_2.5_-associated PAH and BaP_eq_ concentrations at all elevations were significantly increased in winter. In the night, the correlations between the PM_2.5_-associated PAH concentrations and atmospheric temperatures became negatively stronger, notably at lower elevations (r = − 0.73 ~ − 0.86), whereas the BaP_eq_ during daytime and nighttime were not changed significantly in most months. The PAHs analysis with different PM sizes demonstrated the importance of smaller particles such as PM_2.5_. The meteorological variation was more important than elevation to influence the low-elevation PM_2.5_-associated PAH and BaP_eq_ concentrations in an urban area like Kaohsiung City, as the two concentrations were dominated by the PAHs with HMWs and those 5-ring species, respectively.

## Introduction

Polycyclic aromatic hydrocarbons (PAHs) are produced during incomplete combustion of organic compounds such as fossil fuels under anoxic conditions, as their formation was also observed during pyrolysis at high temperatures^1–3^. Works of literature have confirmed that PAHs and their derivatives are carcinogenic and mutagenic to the human body and are harmful to the ecosystem in the environment^4,5^. While many PAHs are present in the environment, the U.S. Environmental Protection Agency (USEPA) and the European Commission have listed 16 PAHs in their priority pollutant lists^6,7^. The International Agency for Research on Cancer (IARC) has classified certain PAH species, namely benzo[a]anthracene (BaA), benzo[a]pyrene (BaP), and dibenzo[a,h]anthtracene (DBA), as probable carcinogens and benzo[b]fluoranthene (BbF), benzo[k]fluoranthene (BkF), and indeno[1,2,3-cd]pyrene (IP) as possible carcinogens to humans^8^.

Particulate matter (PM) is well known as hazardous air pollutants associated with carcinogenicity and other serious health symptoms such as headaches, nausea, and damages to the liver and kidney by inhalation^9^. Typically, particles with a size larger than 10 μm are deposited almost exclusively in the trachea (upper throat) or bronchi region of a human body and are excreted through coughing, sneezing, and running nose. However, particles with sizes from 5 to 10 μm can deposit in the trachea or bronchial area of a human body, as the particles from 1 to 5 μm and those less than 0.1 μm deposit in the gas exchange area of the lung and through the alveolar epithelial cells, eventually circulating throughout the bloodstream and affecting cardiovascular and respiratory systems^10,11^. The occurrences of PAHs on the surface of PM have been frequently reported in previous publications^12,13^. While the PAH concentration distribution and the toxicity could vary in the gas and particle phases, the challenge of identifying the authentic impact of PAH pollution becomes more complex due to PM emission^14^.

PAH and fine particular matter (PM_2.5_) are both critical air pollutants in Taiwan and many countries. The sources of these two hazardous air pollutants include both stationary and mobile sources. For example, vehicular emissions such as diesel and gasoline combustion resulted in high ambient PAH concentrations in a major metropolitan area in Brazil^15^. In central Taiwan, the mean of the BaP-equivalent (BaPeq) concentrations determined by samplings at several stationary sources including the steel and iron industries was 1020 μg m^−3^, while the mean concentrations by analyzing the vehicular exhausts and ambient air at highway toll stations ranged from 8280 to 12,300 ng m^−3^^16^. It was suggested by the epidemiological evidence that PM_2.5_ is associated with increased morbidity and mortality due to respiratory diseases such as asthma, chronic obstructive pulmonary disease, and lung cancer^17^. More importantly, PAHs can be adsorbed onto the surface of PM_2.5_ given their limited vapor pressures and polarity, changing their fates in the environment and influences on the public exposure and health impacts^5,18^. A close correlation between the PAH and PM concentrations was reported in a case study that focused on the burning of three different types of diesel^19^.

Due to their ubiquitousness in the environment, PM_2.5_-associated PAHs are present at different elevations and penetrate buildings through windows, doors, cracks, and ventilation systems, becoming critical sources of PAH exposure in urban areas. Studies have reported that vehicular PM_2.5_ emission near the ground level in metropolitan areas affected the air quality in buildings, notably those near highways and main streets^20,21^. The elevation effect on the PM size distribution is also reported^22^. Considered that human activities mostly occur below building heights, the objective of this study was to investigate the temporal variations of low-elevation PM_2.5_-associated PAH concentrations of a representative high-rise building in Kaohsiung City of southern Taiwan. The data were further used to estimate the BaP_eq_ concentrations by using the toxic equivalent factors (TEFs) (Table S1 in Supporting Information)^23^. The seasonal, monthly, and diurnal variations of the PM_2.5_-associated PAH and BaP_eq_ concentrations were compared for discussion of their pollutions at different low elevations in an urban area. The PAHs associated with different particles sizes that ranged from below 1 (PM_1_) to total suspended particles (TSP) is another focus of this study to understand the particle size effect on PAH distribution, providing additional information for the future management of finer PMs that could be more challenging and hazardous.

## Material and methods

### Study site

**Figure 1:**
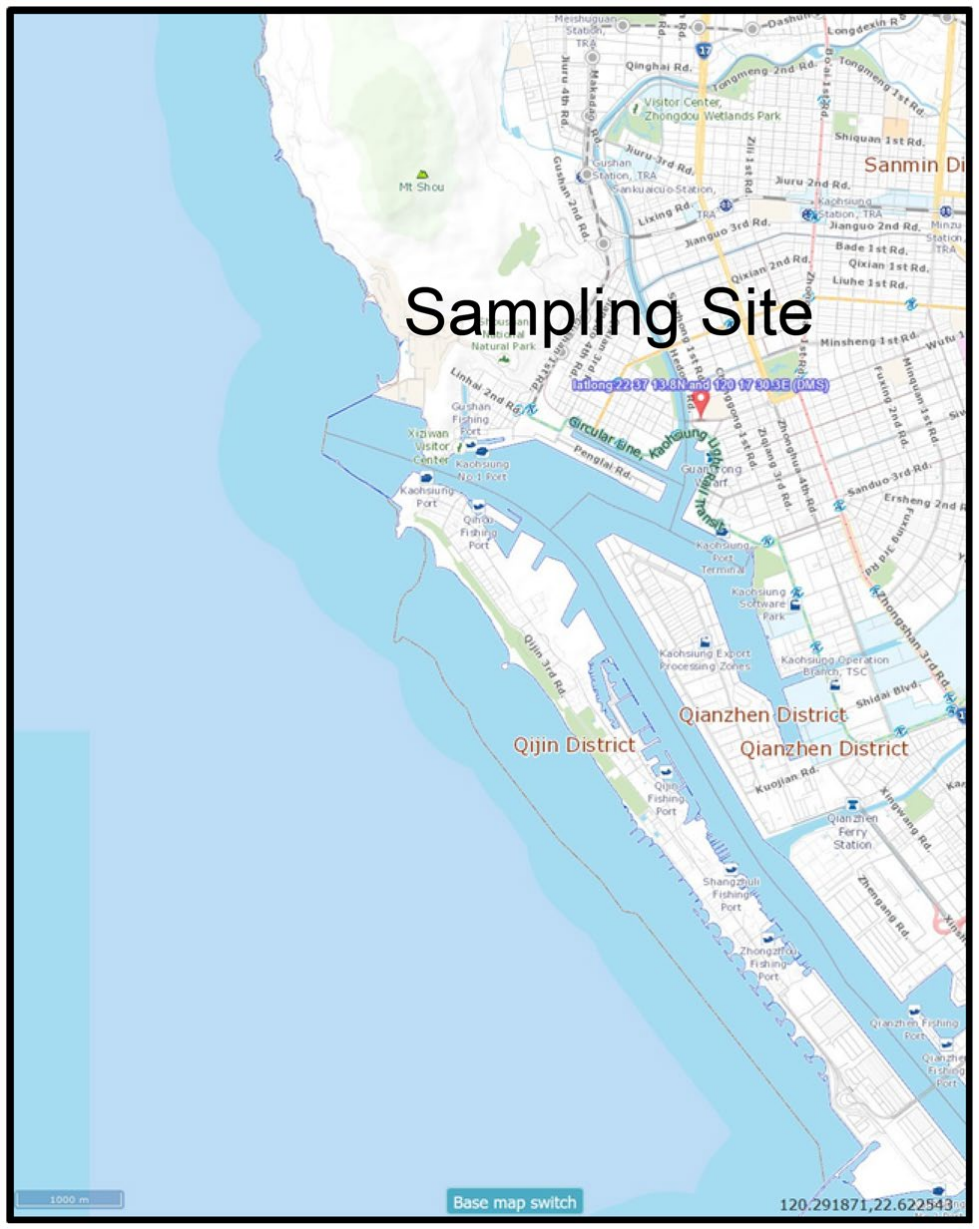
Sampling site selected in this study (created by using Taiwan Map Service, https://maps.nlsc.gov.tw/).

Kaohsiung City is the largest industrial city in southern Taiwan and plays a critical role in benefiting the economy of the nation. The city has a coastal area of 2952 km^2^ and a population of more than 2.7 million, making it the third most populous administrative division and second-largest conurbation in Taiwan. The extent of air pollution in Kaohsiung City due to a great number of pollution-intensive industrial and vehicular activities has been widely known by the public. In this study, a representative nine-story building (22°37′13.8″N, 120°17′30.3″E) in the downtown of Kaohsiung City was selected as the study site. The location of the sampling was near the mouth of Love River, which is one of the largest rivers running from the north of the city to the south. The representativeness of the site was expressed by its location at the city center and its traffic loading (433–633 car h^−1^) near the average traffic loading of the city (709 car h^−1^) (https://www.tbkc.gov.tw/Achievement/ETransport/abc97). Figure 1 illustrates the location of the sampling site in this study.

Table 1 lists the meteorological information of the study site during the sampling periods. The analysis of variance (ANOVA) showed that the ambient temperature, relative humidity, wind speed, and atmospheric pressure were significantly different between daytime and nighttime (p < 0.05). The ambient temperature and atmospheric pressure during daytime or nighttime were also significantly different between different seasons (fall, winter, and spring correspond to the months of September–November, December–February, and March–May, respectively) (p < 0.05).

**Table 1:** Meteorological information of the study site during the sampling periods.

	Temperature (℃)		Relative Humidity (%)		Wind speed (m s^−1^)		Wind direction (degree)^a^		Pressure (hPa)	
	D^b^	N^b^	D	N	D	N	D	N	D	N
Sep	31.0	29.5	73.8	77.3	2.9	1.8	253.6	333.2	1009.6	1011.2
Oct	27.0	24.8	66.9	90.5	1.7	2.2	203.1	165.1	1009.8	1010.5
Nov	26.2	23.3	71.3	85.8	2	2.4	279.5	264.4	1012.0	1011.4
Dec	19.9	16.5	64.2	77.2	3.5	2.9	293.3	327	1019.1	1022.0
Jan	22.2	18.2	70.3	84.8	2.4	1.8	251.5	317.6	1010.8	1011.7
Feb	23.5	19.8	69.4	78.2	3.2	2.4	286.2	260.7	1009.7	1011.9
Mar	22.1	16.3	58.0	58.9	4.3	2.9	296.7	318.2	1010.5	1012.3
Apr	30.2	27.7	71.4	87.5	3.1	1.8	193.5	205.2	1005.8	1006.7
May	30.6	29.0	70.0	77.1	3.4	1.7	292.4	248.7	1007.0	1007.6

^a^The angle measures clockwise rotation.

^b^D and N denote daytime and nighttime, respectively.

### PM sampling

Two different methods were used for sampling of the PM in this study. In the first method^24,25^, a portable air sampler (PEM, SKC XR5000) that utilized a 37 mm quartz fiber filter was used for the collection of PM_2.5_. The flow rate was 4 L min^−1^. The sampling of PM_2.5_ was done once per month from September 2017 to May 2018. The PM_2.5_ was sampled in fine weather to limit the atmospheric interference. The samples were collected at four different heights (floors) including 6.1 (2nd floor), 12.4 (4th floor), 18.4 (6th floor), and 27.1 m (9th floor), expressing the air quality at different low elevations. The works were carried out from 8 am to 8 pm and from 8 pm to 8 am to represent the data during daytime and nighttime, respectively. The locations of the samplings were controlled at least 1 m above the ground of that floor and at least 1 m from adjacent walls to avoid possible interferences. In the second method^25^, PM with different sizes including those with diameters less than 1.0, from 1.0 to 2.5, from 2.5 to 10, from 10 to 18, and larger than 18 μm were sampled. A micro-orifice uniform deposit impactor (MOUDI, Model 100-S4) that utilized 47 mm quartz fiber filters was used. The flow rate was 30 L min^−1^. The sampling was done twice per month from November 2017 to May 2018. Each sampling lasted for 5 days.

### PAH analysis

The PAH concentrations on PM_2.5_ collected in this study were analyzed^26–28^. Before analysis, glassware was washed by using neutral cleaning foam and tap water as well as rinsed with deionized water, followed by heating at 450 °C for 4 h to remove organic residuals. Alumina and quartz fiber filters were heated at 550 °C for 4 h and cooled at room temperature in a desiccator before use. Quartz fiber filters were dried in an oven with a relative humidity of 38–42% for 48 h and then weighed. Sodium sulfate anhydrous was pre-treated by Soxhlet extraction using acetone and n-hexane (1:1 v/v) for 24 h, followed by vacuum drying at 60 °C and heating at 150 °C for 12 h before use.

To analyze the PM_2.5_-associated PAH concentrations, the filter samples were Soxhlet extracted with dichloromethane as the solvent. Before extraction, a mixture of four perdeuterated PAHs including naphthalene-d_8_, fluorene-d_10_, fluoranthene-d_10_, and perylene-d_12_ was added in solvents as surrogates. The extraction time was 24 h. The extracts were concentrated to 0.5 mL by using a rotary evaporator and nitrogen blowdown. Gas chromatography (GC, Agilent 6890 N) coupled with mass spectrometry (MS, Agilent 5973 N) was used to analyze the PAH concentrations in the extracts. Two μL of the extract was injected in the splitless mode. The temperature of the inlet was 310 °C. The GC was equipped with a 30 m × 0.25 mm I.D. Agilent HB-5MS capillary column with 0.25-μm film thickness. After the injection, the oven temperature was programmed as follows: the initial temperature was 50 °C, increased at 10 °C min^−1^ to 280 °C, and then increased at 5 °C min^−1^ to 310 °C. The carrier gas was helium. The flow rate was 1 mL min^−1^. The MS was operated in the electron impact mode. The interface temperature was 310 °C. Prior to the instrumental analysis, a mixture of perdeuterated PAHs that contained acenaphthene-d_10_, phenanthrene-d_10_, benzo(a)anthracene-d_12_, and benzo(a)pyrene-d_12_, and benzo[g,h,i]perylene-d_12_ was added in the extract as the internal standard.

### Quality control and quality assurance

Blanks were carried out to minimize the potential background interferences and noises in each experiment. By following the USEPA’s method detection limit (MDL) procedure (40 CFR 136, Appendix B), Table S2 in Supporting Information lists the MDLs of 16 PM_2.5_-associated PAHs analyzed in this study. The overall recoveries of the PM_2.5_-associated PAH concentrations were 56.4 ± 16.8% (naphthalene-d_8_), 62.7 ± 16.9% (fluorene-d_10_), 68.1 ± 20.8% (fluoranthene-d_10_), and 76.7 ± 14.5% (perylene-d_12_). The recoveries of different PM_2.5_-associated PAH concentrations in the samples collected during daytime ranged from 43.7 ± 11.7% to 80.9 ± 12.1%, while the recoveries of the samples collected during nighttime ranged from 34.3 ± 25.5% to 76.7 ± 14%. A default value of ½ the MDL was used to represent non-detected concentrations for calculations of the mean and standard deviations. Additional information regarding the procedures of extracting and quantifying the PAH concentrations on PM surfaces described here is available in our previous study^24^.

## Results and discussion

### PM_2.5_, PM_2.5_-associated PAH, and BaP_eq_ analyses

**Figure 2:**
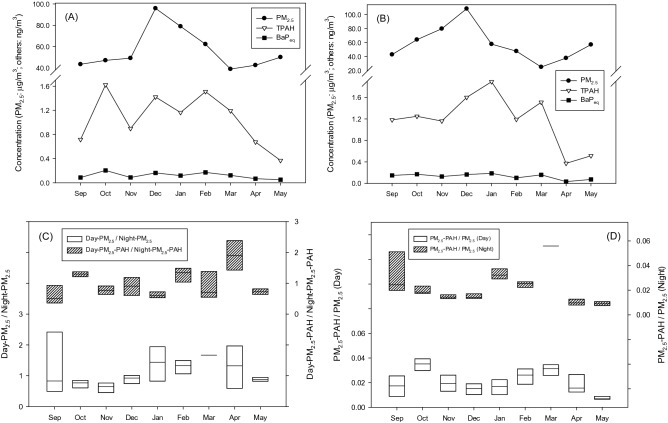
(**A**) Concentrations of PM_2.5_, PM_2.5_-associated PAH, and PM_2.5_-associated BaP_eq_ during daytime and (**B**) nighttime, (**C**) daytime/nighttime PM_2.5_ and PM_2.5_-associated PAH ratios, and (**D**) PM_2.5_-associated PAH/PM_2.5_ ratios during daytime and nighttime.

Before the discussion of the elevation effect, the monthly averages of the PM_2.5_, PM_2.5_-associated PAH, and PM_2.5_-associated BaP_eq_ concentrations detected from all the elevations at the study site were firstly illustrated, respectively (Fig. 2A,B). From the perspective of PM_2.5_, the concentrations were relatively higher during both daytime and nighttime in winter. The maximum PM_2.5_ concentrations during the day (95.9 μg m^−3^) and night (108.4 μg m^−3^) both occurred in winter. Although the correlation analysis indicated a weak correlation between the atmospheric temperature (Table 1) and PM_2.5_ concentrations (r = − 0.40), the variation in PM_2.5_ concentrations in different months was potentially attributable to low temperature and poor mixing circumstances favoring the development of a strong surface temperature inversion.

From the viewpoint of the total PM_2.5_-associated PAHs concentrations, the variations in the day and night were slightly different from those of the PM_2.5_ concentrations (Fig. 2A,B). The concentrations of 16 individual PM_2.5_-associated PAHs detected during daytime and nighttime were given in Tables S4 and S5 in Supporting Information, respectively. The maximum PM_2.5_-associated PAH concentrations in the daytime and nighttime were 1.62 and 1.89 ng m^−3^ occurred in October and January, respectively.

To understand whether the PM_2.5_ and PM_2.5_-associated PAH concentrations exhibited similar variation patterns in the daytime and nighttime, the monthly variations of the daytime/nighttime PM_2.5_ and PM_2.5_-associated PAH ratios were calculated (Fig. 2C). The ANOVA and correlation analysis indicated no significant difference and the limited correlation between the daytime/nighttime ratios of the PM_2.5_ and PM_2.5_-associated PAH ratios (p = 0.11 and r = 0.01), respectively. However, a correlation coefficient of -0.75 indicated a strong negative correlation between the total PM_2.5_-associated PAH concentrations and atmospheric temperature during the sampling period. In comparison with the PM_2.5_ result, the temperature variation seemed to be more critical for affecting the PM_2.5_-associated PAHs concentrations. To understand the potential diurnal effect on the relationship between the PM_2.5_-associated PAH and PM_2.5_ concentrations, the monthly variations of the PM_2.5_-associated PAH/PM_2.5_ ratios during daytime and nighttime were estimated (Fig. 2D). The PM_2.5_-associated PAH/PM_2.5_ ratios ranged from 8.44 × 10^–3^ to 7.55 × 10^–2^ and were not significantly different (p = 0.71) and limitedly correlated (r = 0.20) between daytime and nighttime.

### Seasonal variations at different elevations

The consequence of difficultly characterizing the PM_2.5_-associated PAH concentrations from the perspectives of the PM_2.5_ and meteorological variations could be associated with the neglect of different PAH species on the PM_2.5_. Table 2 lists the contributions of 16 individual PAHs to the total PM_2.5_-associated PAH concentrations during the sampling period. The results showed that BghiP dominated the overall observation, with an average concentration of 0.27 ± 0.04 ng m^−3^ (Tables S3 and S4 in Supporting Information) and 24.68% contribution (Table 2). Note that the contribution percentage increased as the number of the aromatic ring in the PAH increased (Table 2). The higher contribution of the PAHs with high ring numbers was associated with their moderate vapor pressures as well as high organic carbon–water partition coefficients (K_OC_) and octanol–water partition coefficients (K_OW_). Studies that investigated the PAH profiles in urban PM_2.5_ have reported the large abundance of PAHs with a higher number of rings in the particle phase^29,30^, as these species are known to be more indicative of potential health risks posed by the PAHs associated with PMs^13^. Compared to the concerns for the PM_2.5_ and its total PAHs, the distribution of different PAH species was one factor that should not be overlooked.

**Table 2:** Contributions of 16 PAHs with different ring numbers to the total PM_2.5_-associated PAH and BaP_eq_ concentrations observed at different elevations during the sampling periods.

Ring number	Species	PM_2.5_-PAH		BaP_eq_	
		Individual (%)	Total (%)	Individual (%)	Total (%)
2	Nap	0.00	0.00	0.00	0.00
3	Aceny	0.23	10.20	0.00	0.22
	Acen	0.36		0.00	
	Fluo	1.65		0.02	
	Ph	6.48		0.06	
	An	1.48		0.14	
4	Flt	7.31	23.96	0.07	3.37
	Py	6.03		0.06	
	BaA	2.79		2.53	
	Chry + TriPhe	7.83		0.72	
5	BbF	11.39	28.77	10.40	82.78
	BkF	10.24		9.31	
	BaP	5.12		46.70	
	DBA	2.02		16.38	
6	IP	12.64	37.07	11.43	13.64
	BghiP	24.43		2.21	

The corresponding concentration of BaP for a theoretical lifetime cancer risk of 10^–6^ in the air, suggested by the World Health Organization (WHO), is 1 ng m^−3^^23^. The PAH data analyzed in this study was further calculated by using the TEF in Table S1) and discussed from the viewpoint of the PM_2.5_-associated BaP_eq_ concentrations. The variation trends of the monthly averaged PM_2.5_-associated BaP_eq_ concentrations during the sampling period were presented in Fig. 2A and B, respectively. The concentrations of 16 individual PM_2.5_-associated BaP_eq_ detected during daytime and nighttime were given in Tables S6 and S7 in Supporting Information, respectively. The maximum BaP_eq_ concentrations during daytime and nighttime were 0.20 (October) and 0.19 ng m^−3^ (January), respectively, and occurred in the same months when the highest total PM_2.5_-associated PAH concentrations were detected. However, the limited variation in the BaP_eq_ concentrations diminished its negative relationship with the atmospheric temperature (r = − 0.57). In Table 2, BaP became the largest contributor to the PM_2.5_-associated BaP_eq_ concentrations (46.70%). Although the concentrations of the PAHs with higher ring numbers were higher, the BaP_eq_ concentrations were dominated by those 5-ring species with high TEFs including BbF (10.40%), BkF (9.31%), BaP, and DBA (16.38%).

### Seasonal variations at different elevations

**Figure 3:**
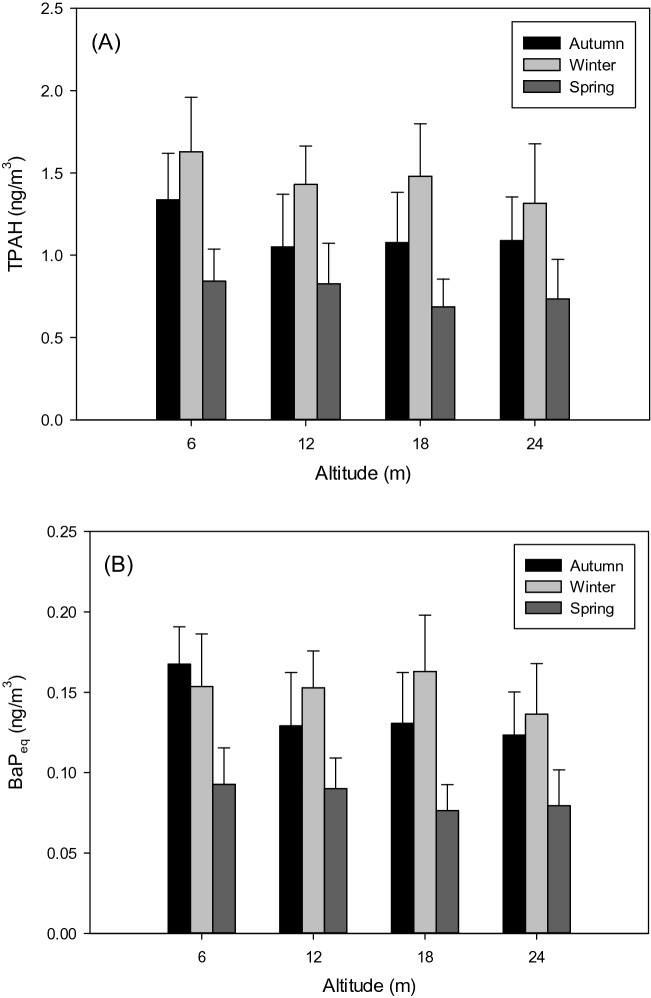
Concentrations of (**A**) PM_2.5_-associated PAH and (**B**) BaP_eq_ in different seasons.

Figure 3A shows the PM_2.5_-associated PAH concentrations detected during daytime and nighttime at the study site in different seasons. The results indicated that PM_2.5_-associated PAH concentrations were significantly increased in winter at four elevations (p < 0.05). The maximum concentration was 1.63 ± 0.33 μg m^−3^ and observed at an elevation of 6 m in winter. Among the observations in different seasons, the highest concentrations in three seasons all occurred at 6 m elevation, potentially attributed to the sources such as vehicular emissions near the study site. Although PM_2.5_ is expected to remain suspended in the air and travel to different elevations of the site, low elevations near the traffic could still result in elevated exposure to PM_2.5_ and hazardous pollutants on the surface. The variation in the PM_2.5_-associated BaP_eq_ concentrations among different seasons was similar to that of the PM_2.5_-associated PAH concentrations (Fig. 3B). The highest BaP_eq_ toxicity (0.17 ± 0.02 ng m^−3^) was detected in autumn at an elevation of 6 m. Nevertheless, the average BaP_eq_ concentrations in winter were relatively higher than those in the other two seasons (p < 0.05). The effect of elevation was negligible on the BaP_eq_ concentration. Overall, in consideration of the potential elevation effect, the seasonal impact of meteorological factors was more important on the variation in the PM_2.5_-associated PAH and BaP_eq_ concentrations. Additional discussions between the effects of elevation, monthly/diurnal concentration variations, and particle size are provided below.

### Monthly variations at different elevations

**Figure 4:**
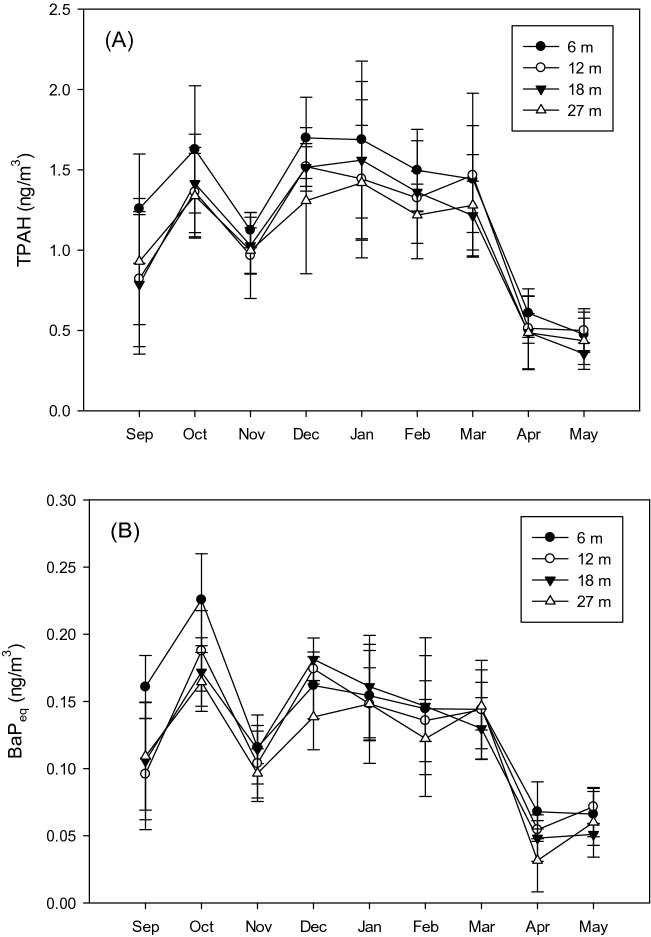
Concentrations of (**A**) PM_2.5_-associated PAH and (**B**) BaP_eq_ in different months.

Figure 4A shows the PM_2.5_-associated PAH concentrations detected at different elevations in different months. The results showed the maximum concentration (1.63 ± 0.40 ng m^−3^) at an elevation of 6 m in October. The monthly variations in the PM_2.5_-associated PAH concentrations were similar among different elevations (r = 0.95–0.98) (the concentrations were increased in October and three months of winter; Fig. 4A). Note that the concentrations at 6 m elevation seemed to be higher particularly when the PM_2.5_-associated PAH concentrations were elevated. The meteorological factors including temperature, wind speed, and relative humidity in different months are given (Fig. S1 in Supporting Information). The temperature was relatively lower at night from December to March, whereas the temperature was elevated in the daytime of April and May. The pattern was similar to those of PM_2.5_ and PAH (Fig. 4). A low atmospheric temperature could reduce the mixing height and limit the vertical movement of pollutants^31^. Table S3 (Supporting Information) lists the Kendall τ between the meteorological parameters and PM_2.5_/PM_2.5_-associated PAH concentrations at different elevations. A significant negative correlation (p < 0.05) was found between the PAH concentrations and temperature at all elevations.

Figure 4B shows the data of the PM_2.5_-associated BaP_eq_ concentrations, with the highest concentration of 0.23 ± 0.03 ng m^−3^ detected in October at an elevation of 6 m. Albeit it was not observed in Fig. 2A and B, the PM_2.5_-associated PAH and BaP_eq_ concentrations were strongly correlated at different elevations. The correlation coefficients between the PM_2.5_-associated PAH and BaP_eq_ concentrations were 0.87, 0.93, 0.97, and 0.96 at elevations of 6, 12, 18, and 24 m, respectively. The Kruskal–Walls test was further used to identify the effects of elevation and monthly variation on the PM_2.5_/PM_2.5_-associated PAH concentrations. The monthly variation of the PM_2.5_/PM_2.5_-associated PAH concentrations were more significant (p < 0.01) compared to the changes of PM2.5 (p = 0.67) and PM2.5-associated PAH (p = 0.45). The monthly variation of meteorological conditions was more important than the elevation to determine the PM_2.5_-associated PAH and BaP_eq_ concentrations. Additional discussions regarding the comparisons between the effects of elevation and other factors such as different time intervals, PAH species, and particle size are given in the following sections.

### Diurnal variations at different elevations

**Figure 5:**
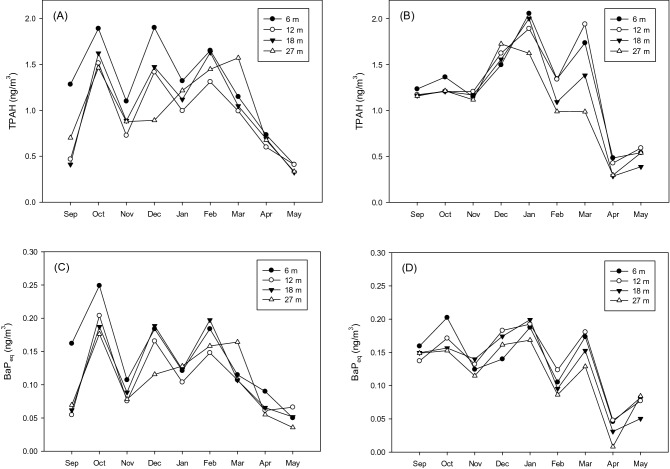
(**A**) Concentrations of PM_2.5_-associated PAH concentrations during daytime and (**B**) nighttime and (**C**) BaP_eq_ concentrations during daytime and (**D**) nighttime in different months.

The monthly variations at different elevations were further divided into the data in the day and night. Figure 5A and B show the averaged PM_2.5_-associated PAH concentrations detected during daytime and nighttime during the monitoring period, respectively. In Fig. 5A, the concentration variations during daytime at different elevations were similar to that of the overall PM_2.5_-associated PAH concentration in Fig. 2A, with relatively higher concentrations being observed in October, December, and February. Note that the daytime concentrations at 6 m elevation were higher in these months (the PM_2.5_-associated PAH concentrations during daytime in October, December, and February were 1.89, 1.90, and 1.65 ng m^–3^). The increase of the PM_2.5_-associated PAH concentrations at a lower elevation could be more critical during the daytime, potentially attributed to more sources such as vehicular emissions in the day. However, Fig. 5B showed different trends of the PM_2.5_-associated PAH concentration variations during nighttime. The concentrations in the night were elevated in winter, followed by significant drops in Spring. The highest concentration (2.1 ng m^−3^) during nighttime occurred at 6 m elevation in January. Table 3 lists the correlation coefficients between the PM_2.5_-associated PAH concentrations and atmospheric temperatures during daytime and nighttime. The analyses suggested stronger negative correlations between the PM_2.5_-associated PAH concentrations and atmospheric temperatures during nighttime, notably at low elevations. The correlation coefficients for the data observed at 6 and 12 m elevations were decreased from − 0.61 and − 0.73 in the day to − 0.78 and − 0.86 in the night, respectively.

**Table 3:** Correlation coefficients of the PM_2.5_-associated PAH or BaP_eq_ concentrations and atmospheric temperatures during daytime and nighttime.

Elevation (m)	PM_2.5_-PAH		BaP_eq_	
	D	N	D	N
6	− 0.61	− 0.78	− 0.31	− 0.42
12	− 0.73	− 0.86	− 0.57	− 0.72
18	− 0.73	− 0.73	− 0.69	− 0.60
24	− 0.63	− 0.63	− 0.67	− 0.44

The PM_2.5_-associated BaP_eq_ concentrations observed during daytime and nighttime were exhibited in Fig. 5C and D, respectively. Similar to Fig. 5A, the highest BaP_eq_ concentrations during daytime occurred at 6 m elevation in October (0.24 ng m^−3^) and gradually decreased. However, the trends of variations in the BaP_eq_ concentrations in the night were slightly different. While the PM_2.5_-associated PAH concentrations at different elevations were increased during nighttime in winter, the BaP_eq_ concentrations in the night were negligibly changed until the drops in April.

**Figure 6:**
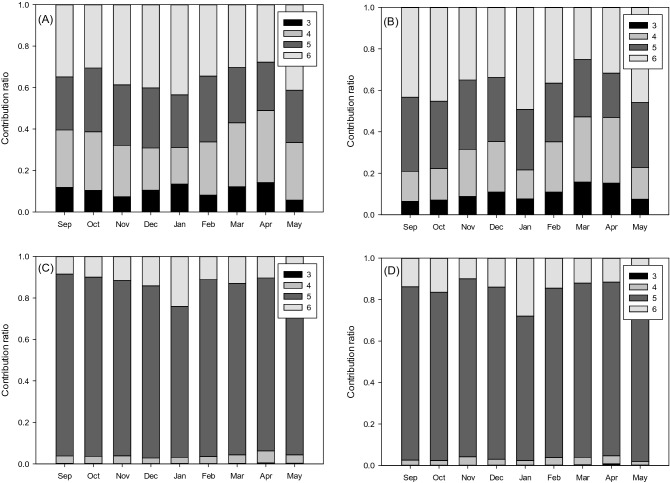
Contributions of PAHs with different ring numbers to the PM_2.5_-associated PAH concentrations during (**A**) daytime and (**B**) nighttime and PM_2.5_-associated BaP_eq_ concentrations during (**C**) daytime and (**D**) nighttime in different months.

Figure 6 shows the contributions of PAHs with different ring numbers to total PM_2.5_-associated PAH and BaP_eq_ concentrations during daytime and nighttime in different months. It was shown that the contributions of those PAHs with high ring numbers dominated the total PM_2.5_-associated PAH concentrations and varied through the months (Fig. 6A and B). However, in Fig. 6C and D, the PAHs with 5 rings including BbF, BkF, BaP, and DBA dominated the PM_2.5_-associated BaP_eq_ concentrations and limitedly changed through the monitoring period. The high toxicities of the 5-ring PAHs were more critical than the temporal variation of the PAH concentration or the elevation effect to determine the PM_2.5_-associated BaP_eq_ concentration.

### Importance of finer PM-associated PAH

**Figure 7:**
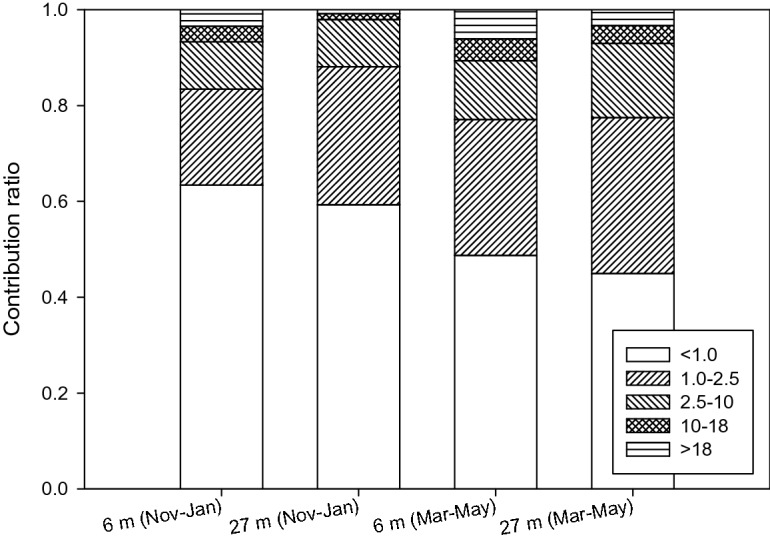
Contributions of the PAH concentrations on the PM with different sizes to total PM-associated PAH concentrations in different samples.

The focus of this study was the PM_2.5_-associated PAH concentrations and their toxicity. To investigate the contributions of PAHs on PMs with other different sizes, PMs including TSP, particles equal to or smaller than 10 μm (PM_10_) and PM_1_ were sampled, followed by analyses of the PM-associated PAH concentrations, as shown in Fig. 7. The result showed that the contributions to total PM-associated PAH concentrations were dominated by the PAHs associated with the smaller PMs. In winter, the contributions of the PM_2.5_- and PM_1.0_-associated PAH concentrations were 83–88% and 59–63%, as the numbers were dropped to 77–78% and 45–49% in summer, respectively. The finding suggested the critical impact of the PAHs associated with smaller particle sizes such as PM_2.5_ and PM_1_. The finding suggested the critical impact of the PAHs associated with smaller particle sizes such as PM_2.5_. In comparison with the elevation effect, the seasonal variation seemed to be more important for affecting the PM-associated PAH concentrations.

### Comparison with other studies

While the PM_2.5_ concentration shown in Fig. 2 mostly exceeded the standards by the USEPA (primary and secondary annual average standards with levels of 12.0 and 15.0 µg m^−3^, respectively; 24-h standards with 98th percentile forms and levels of 35 µg m^−3^)^9^ and Taiwan EPA (yearly average and 24-h value of 15 and 35 µg m^−3^, respectively)^32^, the PM_2.5_-associated PAH concentrations observed in this study were compared to those reported in publications (Table 4). These countries or regions were selected given their metrological conditions and characteristics similar to that of the sampling site of interest in this study. The results showed that the PM_2.5_-associated PAH concentrations detected in this study were similar to those reported by Yang et al. that investigated the concentrations in Hsinchu City that houses Hsinchu Science Park, a major semiconductor manufacturing region in Taiwan. However, the concentrations were relatively lower than the numbers in New York in U.S.^33^, Singapore^20^, and Bangkok in Thailand Pongpiachan^34^. The concentration differences between these observations suggest the impact of different degrees of city development on the PM_2.5_-associated PAH concentrations. Note that Jung et al. reported limited vertical variation of PM_2.5_ concentration since the influence of the regional atmosphere was more important than that of sources near the ground. However, Kalaiarasan et al. have reported that the PAH concentrations were elevated at lower elevations and gradually decreased as the elevation was increased.

**Table 4:** Comparison of our and other studies analyzing the PM_2.5_-associated PAH concentrations.

Study	Year	Site location	Concentration (ng m^−3^)	Elevation (m)
Yan et al.^30^	2017	Hsinchu, Taiwan	1.76 ± 1.98	21
Jung et al.^28^	2011	New York, U.S.	1.96 ± 1.35	0–6
			2.45 ± 2.21	7–15
			1.84 ± 1.45	> 16
Kalaiarasan et al.^20^^a^	2009	Singapore	2.70 ± 1.23	11
			4.54 ± 2.06	28
			3.67 ± 1.53	39
Kalaiarasan et al.^20^^b^	2009	Singapore	6.79 ± 2.99	11
			6.71 ± 2.36	28
			4.67 ± 1.52	47
Pongpiachan^29^^c^	2013	Bangkok, Thailand	3.08 ± 1.92	38
			4.79 ± 4.62	158
			2.01 ± 1.27	328
Pongpiachan^29^^d^	2013	Bangkok, Thailand	6.45 ± 2.74	38
			4.88 ± 4.38	158
			2.28 ± 0.91	328

^a^The study was carried out at a building of point block configuration.

^b^The study was carried out at a building of slab block configuration.

^c^The study analyzed the concentrations during the daytime.

^d^The study analyzed the concentrations during the nighttime.

## Conclusion

As previous studies have investigated the pollutant concentration distributions at elevations typically above one to a few hundred meters^22,35–37^, this study investigated the vertical distribution of PAHs at low elevations (< 30 m) considered the human activities mostly occur below building heights. The seasonal, monthly, and diurnal variations of the PM_2.5_-associated PAH and BaP_eq_ concentrations at four different low elevations in the largest industrial city of southern Taiwan were analyzed. It was shown that, in comparison with the PM_2.5_ observations, temperature variation was more critical to affecting the PM_2.5_-associated PAH concentrations, which were dominated by BghiP and others with high ring number. The PM_2.5_-associated BaP_eq_ concentrations were mainly dominated by those 5-rings species with high TEFs. As to the seasonal variations at different elevations, the PM_2.5_-associated PAH and BaP_eq_ concentrations at all four elevations were significantly increased in winter, with limited differences between the four elevations. Similarly, the monthly temperature variation was more important than elevation to affect the PM_2.5_-associated PAH and BaP_eq_ concentrations. In the night, the correlations between the PM_2.5_-associated PAH concentrations and atmospheric temperatures became negatively stronger, notably at low elevations, whereas the BaP_eq_ concentrations during daytime and nighttime were both dominated by 5-ring PAHs and limitedly changed in most months. The analysis of PAHs associated with different particle sizes demonstrated the importance of the PMs associated with smaller particle sizes such as PM_2.5_. Overall, although several high PM_2.5_-associated PAH and BaP_eq_ concentrations were found at a lower elevation (e.g., 6 m), the temporal variations of meteorological conditions were more important than elevation to influence the PM_2.5_-associated PAH and BaP_eq_ concentrations in an urban area like Kaohsiung City, as the two concentrations were dominated by the PAHs with high molecular weights and those 5-ring species, respectively.

## Supplementary Information

Supplementary Information.

## References

[1] Rengarajan T (2015). Exposure to polycyclic aromatic hydrocarbons with special focus on cancer. Asian Pac. J. Trop. Biomed..

[2] Richter H, Howard JB (2000). Formation of polycyclic aromatic hydrocarbons and their growth to soot: A review of chemical reaction pathways. Prog. Energ. Combust..

[3] Williams PT, Nugranad N (2000). Comparison of products from the pyrolysis and catalytic pyrolysis of rice husks. Energy.

[4] Zhang Y, Tao S (2009). Global atmospheric emission inventory of polycyclic aromatic hydrocarbons (PAHs) for 2004. Atmos. Environ..

[5] Ravindra K, Sokhi R, Van Grieken R (2008). Atmospheric polycyclic aromatic hydrocarbons: Source attribution, emission factors and regulation. Atmos. Environ..

[6] USEPA. *Polycyclic Aromatic Hydrocarbons (PAHs)*, https://www.google.com/url?sa=t&rct=j&q=&esrc=s&source=web&cd=&ved=2ahUKEwjrw53c7vbqAhUbyIsBHX6qBFgQFjABegQIBxAB&url=https%3A%2F%2Fwww.epa.gov%2Fsites%2Fproduction%2Ffiles%2F2014-03%2Fdocuments%2Fpahs_factsheet_cdc_2013.pdf&usg=AOvVaw00o0sxjtVh6BTGHp2df7aB (2008).

[7] Lerda D (2011). JRC Technical Report: Polycyclic Aromatic Hydrocarbons (PAHs) Factsheet.

[8] IARC. *IARC Monographs on the Evaluation of Carcinogenic Risks to Humans*. http://monographs.iarc.fr/ENG/Classification/latest_classif.php (1987). 10.1289/ehp.94102590 PMC1569767 9679121

[9] USEPA. *National Ambient Air Quality Standards (NAAQS) for PM*. https://www.epa.gov/pm-pollution/national-ambient-air-quality-standards-naaqs-pm#rule-summary (2020).

[10] Elmes M, Gasparon M (2017). Sampling and single particle analysis for the chemical characterisation of fine atmospheric particulates: A review. J. Environ. Manage.

[11] Kampa M, Castanas E (2008). Human health effects of air pollution. Environ. Pollut..

[12] He C (2010). Characteristics of polycyclic aromatic hydrocarbons emissions of diesel engine fueled with biodiesel and diesel. Fuel.

[13] Chen WH, Chen GF, Lin YC (2019). Influence of emulsified biodiesel on the emission and health risk of polycyclic aromatic hydrocarbons in the vapor and particulate phases during engine combustion. Environ. Sci. Pollut. Res..

[14] Pehnec G, Jakovljevic I (2018). Carcinogenic potency of airborne polycyclic aromatic hydrocarbons in relation to the particle fraction size. Int. J. Environ. Res. Public Health.

[15] Teixeira EC (2012). Source identification and seasonal variation of polycyclic aromatic hydrocarbons associated with atmospheric fine and coarse particles in the Metropolitan Area of Porto Alegre, RS, Brazil. Atmos. Res..

[16] Chen SC, Liao CM (2006). Health risk assessment on human exposed to environmental polycyclic aromatic hydrocarbons pollution sources. Sci. Total Environ..

[17] Pope CA (2002). Lung cancer, cardiopulmonary mortality, and long-term exposure to fine particulate air pollution. JAMA.

[18] Manoli E, Voutsa D, Samara C (2002). Chemical characterization and source identification/apportionment of fine and coarse air particles in Thessaloniki, Greece. Atmos. Environ..

[19] He Q (2017). Particle dry deposition of polycyclic aromatic hydrocarbons and its risk assessment in a typical coal-polluted and basin city, northern China. Atmos. Pollut. Res..

[20] Kalaiarasan M, Balasubramanian R, Cheong KWD, Tham KW (2009). Particulate-bound polycyclic aromatic hydrocarbons in naturally ventilated multi-storey residential buildings of Singapore: Vertical distribution and potential health risks. Build. Environ..

[21] Zhang Z-H, Khlystov A, Norford LK, Tan Z-K, Balasubramanian R (2017). Characterization of traffic-related ambient fine particulate matter (PM 2.5) in an Asian city: Environmental and health implications. Atmos. Environ..

[22] McKendry IG, Sturman AP, Vergeiner J (2004). Vertical profiles of particulate matter size distributions during winter domestic burning in Christchurch, New Zealand. Atmos. Environ..

[23] Nisbet ICT, Lagoy PK (1992). Toxic equivalency factors (Tefs) for polycyclic aromatic-hydrocarbons (Pahs). Regul. Toxicol. Pharm..

[24] Lee CL (2016). A new grid-scale model simulating the spatiotemporal distribution of PM2.5-PAHs for exposure assessment. J. Hazard. Mater..

[25] Chen SJ, Liao SH, Jian WJ, Lin CC (1997). Particle size distribution of aerosol carbons in ambient air. Environ. Int..

[26] Lai IC, Chang YC, Lee CL, Chiou GY, Huang HC (2013). Source identification and characterization of atmospheric polycyclic aromatic hydrocarbons along the southwestern coastal area of Taiwan: With a GMDH approach. J. Environ. Manage..

[27] Huang HC, Lee CL, Lai CH, Fang MD, Lai IC (2012). Transboundary movement of polycyclic aromatic hydrocarbons (PAHs) in the Kuroshio Sphere of the western Pacific Ocean. Atmos. Environ..

[28] Lai IC, Lee CL, Zeng KY, Huang HC (2011). Seasonal variation of atmospheric polycyclic aromatic hydrocarbons along the Kaohsiung coast. J. Environ. Manage..

[29] Gong XS (2019). Characterization of polycyclic aromatic hydrocarbon (PAHs) source profiles in urban PM2.5 fugitive dust: A large-scale study for 20 Chinese cites. Sci. Total Environ..

[30] Vuong QT, Thang PQ, Nguyen TNT, Ohura T, Choi SD (2020). Seasonal variation and gas/particle partitioning of atmospheric halogenated polycyclic aromatic hydrocarbons and the effects of meteorological conditions in Ulsan. South Korea. Environ. Pollut..

[31] Hsieh M-T (2020). Simulating the spatiotemporal distribution of BTEX with an hourly grid-scale model. Chemosphere.

[32] TWEPA. *Air Quality Standards, Taiwan Air Quality Monitoring Network, Taiwan Environmental Protection Agency (TWEPA)*. https://airtw.epa.gov.tw/ENG/Information/Standard/Rules.aspx (2012).

[33] Jung KH (2011). Effects of floor level and building type on residential levels of outdoor and indoor polycyclic aromatic hydrocarbons, black carbon, and particulate matter in New York City. Atmosphere.

[34] Pongpiachan S (2013). Vertical distribution and potential risk of particulate polycyclic aromatic hydrocarbons in high buildings of Bangkok, Thailand. Asian Pac. J. Cancer Prev..

[35] Moeinaddini M (2014). Source apportionment of PAHs and n-alkanes in respirable particles in Tehran, Iran by wind sector and vertical profile. Environ. Sci. Pollut. R.

[36] Kalberer M, Henne S, Prevot ASH, Steinbacher M (2004). Vertical transport and degradation of polycyclic aromatic hydrocarbons in an Alpine Valley. Atmos. Environ..

[37] Tao S (2007). Vertical distribution of polycyclic aromatic hydrocarbons in atmospheric boundary layer of Beijing in winter. Atmos. Environ..

