# Supplementary material for: Temporal and vertical variations of polycyclic aromatic hydrocarbon at low elevations in an industrial city of southern Taiwan
Source: Sci Rep. 2021 Feb 10;11:3453. doi: 10.1038/s41598-021-83155-7 (PMC7876100; doi:10.1038/s41598-021-83155-7)
Supplement: Supplementary file 1 — Supplementary Information. [file 41598_2021_83155_MOESM1_ESM.docx]

Temporal and vertical variations of polycyclic aromatic hydrocarbon at low elevations in an industrial city of southern Taiwan

Wei-Hsiang Chen^1,2,3^, Ming-Tsuen Hsieh^4^, Jie-Yu You^4^, Adnan Quadir^4^, and Chon-Lin Lee ^2,3,4,5*^

^1^ Institute of Environmental Engineering, National Sun Yat-sen University, Kaohsiung 804, Taiwan

^2^ Aerosol Science and Research Center, National Sun Yat-sen University, Kaohsiung 804, Taiwan

^3^ Department of Public Health, Kaohsiung Medical University, Kaohsiung, Taiwan

^4^ Department of Marine Environment and Engineering, National Sun Yat-sen University, Kaohsiung 804, Taiwan

^5^ Department of Applied Chemistry, Providence University, Taichung, Taiwan

Corresponding author: Chon-Lin Lee, Tel: 886-7-5252000 ext 5066, E-mail address: [linnohc@fac.nsysu.edu.tw](mailto:linnohc@fac.nsysu.edu.tw)

Table S1. Toxic equivalent factors (TEFs) of 16 PAHs used in this study

| Species | NaP | Aceny | Acen | Fluo | Ph | An | Flt | Py |
| --- | --- | --- | --- | --- | --- | --- | --- | --- |
| TEF | 0.001 | 0.001 | 0.001 | 0.001 | 0.001 | 0.01 | 0.001 | 0.001 |
| Species | BaA | Chry+TriPhe | BbF | BkF | BaP | DBA | IP | BghiP |
| TEF | 0.1 | 0.01 | 0.1 | 0.1 | 1 | 1 | 0.1 | 0.01 |
| - Source: Nisbet and Lagoy, 1992 ^1^ - The PAHs include naphthalene (Nap), acenaphthylene (Aceny), acenaphthene (Acen), fluorene (Fluo), phenanthrene (Ph), anthracene (Ant), fluoranthene (Flt), pyrene (Py), benzo[a]anthracene (BaA), chrysene (CHR), benzo[b]fluoranthene (BbF), benzo[k]fluoranthene (BkF), benzo[a]pyrene (BaP), indeno[1,2,3-cd]pyrene (IP), dibenzo[a,h]anthtracene (DBA), and benzo[g,h,i]perylene (BghiP) | | | | | | | | |

Table S2. Method detection limits (MDLs) of 16 PM_2.5_-associated PAHs of interest in this study

| Species | NaP | Aceny | Acen | Fluo | Ph | An | Flt | Py |
| --- | --- | --- | --- | --- | --- | --- | --- | --- |
| MDL (ng/m^3^) | 12.37 | 0.12 | 0.07 | 0.16 | 0.39 | 0.06 | 0.13 | 0.09 |
| Species | BaA | Chry+TriPhe | BbF | BkF | BaP | DBA | IP | BghiP |
| MDL  (ng/m^3^) | 0.07 | 0.07 | 0.04 | 0.04 | 0.09 | 0.04 | 0.04 | 0.10 |
| - The PAHs include naphthalene (Nap), acenaphthylene (Aceny), acenaphthene (Acen), fluorene (Fluo), phenanthrene (Ph), anthracene (Ant), fluoranthene (Flt), pyrene (Py), benzo[a]anthracene (BaA), chrysene (CHR), benzo[b]fluoranthene (BbF), benzo[k]fluoranthene (BkF), benzo[a]pyrene (BaP), indeno[1,2,3-cd]pyrene (IP), dibenzo[a,h]anthtracene (DBA), and benzo[g,h,i]perylene (BghiP) | | | | | | | | |


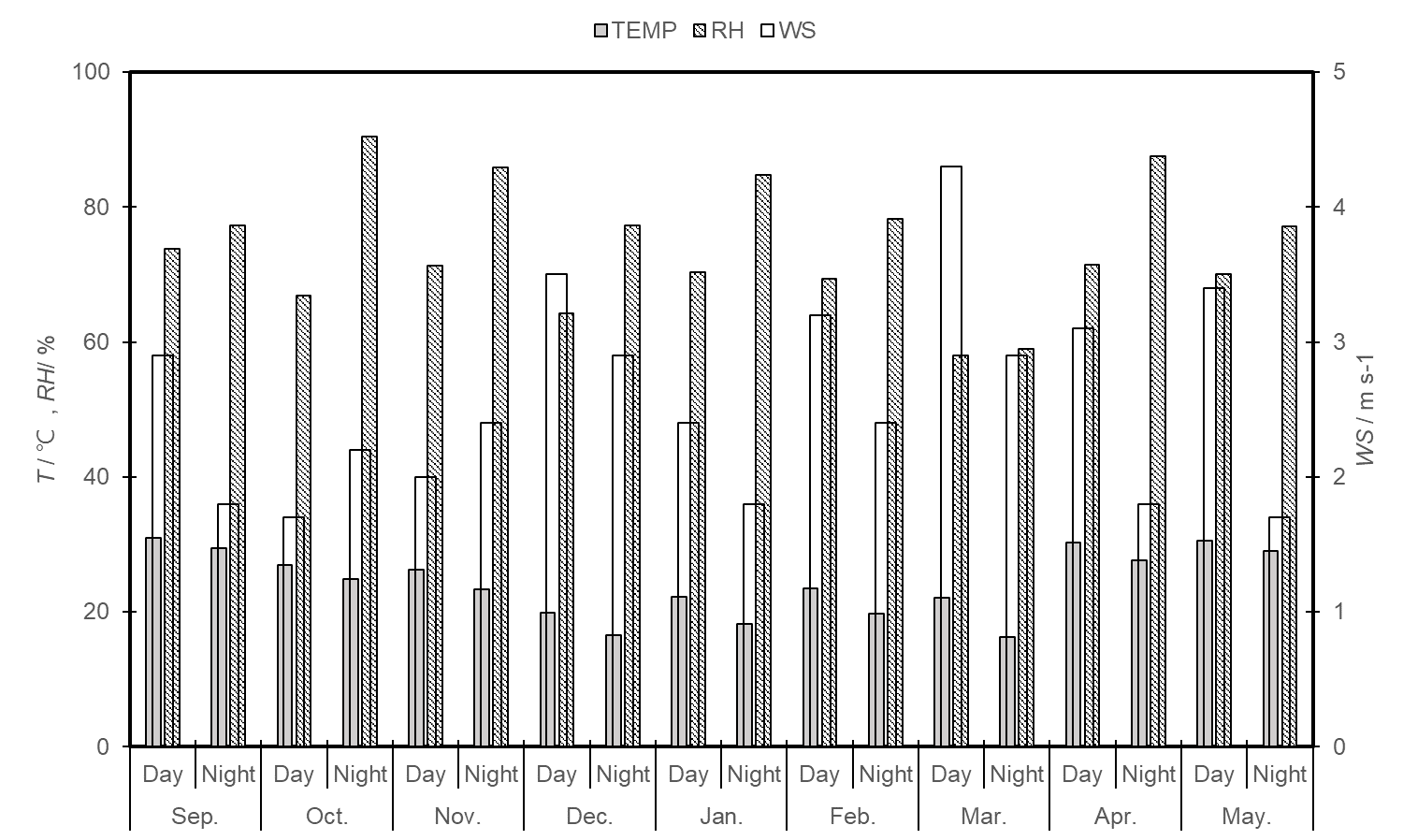


Figure S1. Meteorological factors that include the monthly ambient temperature (T), wind speed (WS), and relative humidity (RH) of the sampling site

Table S3. Kendall τ analyses between the meteorological factors including ambient temperature (temp), relative humidity (RH), wind direction (WD), wind speed (WS), and atmospheric pressure as well as the PM_2.5_ and PM_2.5_-associated PAH concentrations

|  | TEMP | RH | WD | WS | Pressure | 6mPAH | 12mPAH | 18mPAH | 27mPAH | 6mPM | 12mPM | 18mPM | 27mPM |
| --- | --- | --- | --- | --- | --- | --- | --- | --- | --- | --- | --- | --- | --- |
| TEMP | 1.000 | 0.098 | -.346^*^ | -0.169 | -.590^**^ | -.472^**^ | -.621^**^ | -.438^*^ | -.425^*^ | -0.307 | -0.190 | -0.236 | -0.098 |
| RH | 0.098 | 1.000 | -0.229 | -.426^*^ | -0.079 | -0.144 | -0.085 | -0.059 | -0.098 | 0.176 | 0.111 | 0.170 | 0.229 |
| WD | -.346^*^ | -0.229 | 1.000 | 0.250 | .459^**^ | 0.184 | 0.281 | 0.255 | 0.294 | -0.059 | 0.007 | 0.052 | 0.020 |
| WS | -0.169 | -.426^*^ | 0.250 | 1.000 | 0.081 | 0.041 | -0.007 | 0.007 | -0.020 | 0.115 | 0.115 | -0.014 | 0.020 |
| Pressure | -.590^**^ | -0.079 | .459^**^ | 0.081 | 1.000 | .401^*^ | .538^**^ | .433^*^ | 0.275 | 0.249 | 0.157 | 0.230 | 0.302 |
| 6mPAH | -.472^**^ | -0.144 | 0.184 | 0.041 | .401^*^ | 1.000 | .761^**^ | .761^**^ | .525^**^ | 0.315 | 0.039 | 0.322 | 0.105 |
| 12mPAH | -.621^**^ | -0.085 | 0.281 | -0.007 | .538^**^ | .761^**^ | 1.000 | .791^**^ | .516^**^ | 0.216 | -0.007 | 0.302 | 0.190 |
| 18mPAH | -.438^*^ | -0.059 | 0.255 | 0.007 | .433^*^ | .761^**^ | .791^**^ | 1.000 | .621^**^ | 0.320 | 0.072 | .407^*^ | 0.242 |
| 27mPAH | -.425^*^ | -0.098 | 0.294 | -0.020 | 0.275 | .525^**^ | .516^**^ | .621^**^ | 1.000 | 0.307 | 0.137 | 0.289 | 0.124 |
| 6mPM | -0.307 | 0.176 | -0.059 | 0.115 | 0.249 | 0.315 | 0.216 | 0.320 | 0.307 | 1.000 | .647^**^ | .643^**^ | .399^*^ |
| 12mPM | -0.190 | 0.111 | 0.007 | 0.115 | 0.157 | 0.039 | -0.007 | 0.072 | 0.137 | .647^**^ | 1.000 | .525^**^ | 0.333 |
| 18mPM | -0.236 | 0.170 | 0.052 | -0.014 | 0.230 | 0.322 | 0.302 | .407^*^ | 0.289 | .643^**^ | .525^**^ | 1.000 | .577^**^ |
| 27mPM | -0.098 | 0.229 | 0.020 | 0.020 | 0.302 | 0.105 | 0.190 | 0.242 | 0.124 | .399^*^ | 0.333 | .577^**^ | 1.000 |

*: *p*<.05; **: *p*<.01

Table S4. Concentrations of 16 PM_2.5_-associated PAHs detected during daytime

| Species | Average (ng m^-3^) | | | | | | | | |
| --- | --- | --- | --- | --- | --- | --- | --- | --- | --- |
|  | Sep | Oct | Nov | Dec | Jan | Feb | Mar | Apr | May |
| Nap | 0.00 | 0.00 | 0.00 | 0.00 | 0.00 | 0.00 | 0.00 | 0.00 | 0.00 |
| Aceny | 0.01 | 0.00 | 0.00 | 0.00 | 0.00 | 0.00 | 0.00 | 0.00 | 0.00 |
| Acen | 0.00 | 0.00 | 0.00 | 0.00 | 0.00 | 0.00 | 0.00 | 0.01 | 0.00 |
| Fluo | 0.04 | 0.02 | 0.00 | 0.02 | 0.03 | 0.00 | 0.01 | 0.03 | 0.01 |
| Ph | 0.02 | 0.12 | 0.07 | 0.13 | 0.12 | 0.11 | 0.12 | 0.03 | 0.00 |
| An | 0.01 | 0.02 | 0.00 | 0.01 | 0.01 | 0.01 | 0.01 | 0.02 | 0.01 |
| Flt | 0.06 | 0.13 | 0.07 | 0.10 | 0.06 | 0.14 | 0.12 | 0.05 | 0.03 |
| Py | 0.04 | 0.11 | 0.05 | 0.07 | 0.05 | 0.09 | 0.10 | 0.07 | 0.02 |
| BaA | 0.02 | 0.05 | 0.02 | 0.03 | 0.03 | 0.04 | 0.04 | 0.03 | 0.02 |
| Chry+TriPhe | 0.07 | 0.17 | 0.07 | 0.09 | 0.07 | 0.12 | 0.11 | 0.09 | 0.03 |
| BbF | 0.05 | 0.19 | 0.09 | 0.17 | 0.15 | 0.19 | 0.15 | 0.06 | 0.03 |
| BkF | 0.07 | 0.17 | 0.12 | 0.13 | 0.08 | 0.18 | 0.09 | 0.05 | 0.02 |
| BaP | 0.03 | 0.09 | 0.05 | 0.10 | 0.05 | 0.10 | 0.06 | 0.04 | 0.02 |
| DBA | 0.03 | 0.05 | 0.00 | 0.00 | 0.02 | 0.01 | 0.02 | 0.01 | 0.02 |
| IP | 0.05 | 0.17 | 0.07 | 0.19 | 0.26 | 0.16 | 0.14 | 0.06 | 0.04 |
| BghiP | 0.20 | 0.32 | 0.27 | 0.38 | 0.24 | 0.37 | 0.22 | 0.13 | 0.11 |
|  | Standard deviation (ng m^-3^) | | | | | | | | |
| Nap | 0.00 | 0.00 | 0.00 | 0.00 | 0.00 | 0.00 | 0.00 | 0.00 | 0.00 |
| Aceny | 0.03 | 0.00 | 0.00 | 0.00 | 0.00 | 0.00 | 0.00 | 0.01 | 0.00 |
| Acen | 0.01 | 0.01 | 0.00 | 0.00 | 0.00 | 0.00 | 0.01 | 0.00 | 0.00 |
| Fluo | 0.02 | 0.02 | 0.00 | 0.03 | 0.03 | 0.00 | 0.01 | 0.01 | 0.01 |
| Ph | 0.04 | 0.02 | 0.05 | 0.11 | 0.05 | 0.02 | 0.04 | 0.03 | 0.00 |
| An | 0.01 | 0.00 | 0.00 | 0.01 | 0.01 | 0.00 | 0.02 | 0.01 | 0.00 |
| Flt | 0.04 | 0.02 | 0.02 | 0.05 | 0.02 | 0.02 | 0.04 | 0.01 | 0.00 |
| Py | 0.03 | 0.02 | 0.02 | 0.03 | 0.01 | 0.01 | 0.04 | 0.01 | 0.00 |
| BaA | 0.01 | 0.00 | 0.01 | 0.01 | 0.01 | 0.01 | 0.01 | 0.00 | 0.00 |
| Chry+TriPhe | 0.04 | 0.01 | 0.01 | 0.03 | 0.01 | 0.02 | 0.04 | 0.02 | 0.01 |
| BbF | 0.03 | 0.03 | 0.02 | 0.03 | 0.01 | 0.02 | 0.03 | 0.00 | 0.01 |
| BkF | 0.04 | 0.02 | 0.01 | 0.04 | 0.01 | 0.03 | 0.02 | 0.00 | 0.01 |
| BaP | 0.02 | 0.01 | 0.01 | 0.02 | 0.00 | 0.01 | 0.02 | 0.01 | 0.01 |
| DBA | 0.02 | 0.02 | 0.00 | 0.01 | 0.00 | 0.01 | 0.00 | 0.01 | 0.01 |
| IP | 0.03 | 0.03 | 0.01 | 0.02 | 0.02 | 0.03 | 0.02 | 0.01 | 0.00 |
| BghiP | 0.11 | 0.04 | 0.03 | 0.06 | 0.01 | 0.03 | 0.01 | 0.01 | 0.02 |

Table S5. Concentrations of 16 PM_2.5_-associated PAHs detected during nighttime

| Species | Average (ng m^-3^) | | | | | | | | |
| --- | --- | --- | --- | --- | --- | --- | --- | --- | --- |
|  | Sep | Oct | Nov | Dec | Jan | Feb | Mar | Apr | May |
| Nap | 0.00 | 0.00 | 0.00 | 0.00 | 0.00 | 0.00 | 0.00 | 0.00 | 0.00 |
| Aceny | 0.00 | 0.00 | 0.00 | 0.00 | 0.00 | 0.00 | 0.01 | 0.00 | 0.00 |
| Acen | 0.00 | 0.00 | 0.00 | 0.00 | 0.00 | 0.00 | 0.01 | 0.01 | 0.00 |
| Fluo | 0.01 | 0.01 | 0.01 | 0.01 | 0.02 | 0.00 | 0.02 | 0.02 | 0.01 |
| Ph | 0.07 | 0.07 | 0.09 | 0.16 | 0.11 | 0.12 | 0.16 | 0.00 | 0.01 |
| An | 0.00 | 0.01 | 0.00 | 0.00 | 0.02 | 0.01 | 0.03 | 0.02 | 0.01 |
| Flt | 0.04 | 0.04 | 0.08 | 0.16 | 0.07 | 0.10 | 0.17 | 0.03 | 0.02 |
| Py | 0.03 | 0.04 | 0.05 | 0.09 | 0.05 | 0.08 | 0.13 | 0.05 | 0.02 |
| BaA | 0.03 | 0.03 | 0.04 | 0.03 | 0.03 | 0.03 | 0.04 | 0.01 | 0.01 |
| Chry+TriPhe | 0.07 | 0.08 | 0.09 | 0.11 | 0.11 | 0.08 | 0.14 | 0.03 | 0.03 |
| BbF | 0.16 | 0.14 | 0.13 | 0.20 | 0.32 | 0.14 | 0.19 | 0.03 | 0.06 |
| BkF | 0.17 | 0.16 | 0.18 | 0.19 | 0.15 | 0.14 | 0.13 | 0.03 | 0.05 |
| BaP | 0.05 | 0.06 | 0.07 | 0.09 | 0.05 | 0.05 | 0.08 | 0.02 | 0.02 |
| DBA | 0.04 | 0.05 | 0.01 | 0.01 | 0.03 | 0.00 | 0.02 | 0.00 | 0.03 |
| IP | 0.17 | 0.25 | 0.10 | 0.20 | 0.48 | 0.12 | 0.17 | 0.03 | 0.08 |
| BghiP | 0.34 | 0.32 | 0.31 | 0.34 | 0.45 | 0.32 | 0.21 | 0.09 | 0.16 |
|  | Standard deviation (ng m^-3^) | | | | | | | | |
| Nap | 0.00 | 0.00 | 0.00 | 0.00 | 0.00 | 0.00 | 0.00 | 0.00 | 0.00 |
| Aceny | 0.00 | 0.00 | 0.00 | 0.00 | 0.00 | 0.00 | 0.02 | 0.01 | 0.00 |
| Acen | 0.00 | 0.00 | 0.00 | 0.00 | 0.00 | 0.00 | 0.01 | 0.01 | 0.01 |
| Fluo | 0.01 | 0.01 | 0.01 | 0.01 | 0.01 | 0.00 | 0.02 | 0.01 | 0.02 |
| Ph | 0.01 | 0.01 | 0.00 | 0.02 | 0.03 | 0.03 | 0.08 | 0.00 | 0.03 |
| An | 0.01 | 0.01 | 0.01 | 0.01 | 0.01 | 0.01 | 0.01 | 0.00 | 0.00 |
| Flt | 0.00 | 0.00 | 0.01 | 0.01 | 0.02 | 0.03 | 0.09 | 0.01 | 0.01 |
| Py | 0.00 | 0.00 | 0.00 | 0.01 | 0.02 | 0.05 | 0.06 | 0.01 | 0.00 |
| BaA | 0.00 | 0.00 | 0.01 | 0.00 | 0.01 | 0.01 | 0.01 | 0.01 | 0.01 |
| Chry+TriPhe | 0.01 | 0.01 | 0.01 | 0.01 | 0.02 | 0.01 | 0.04 | 0.01 | 0.01 |
| BbF | 0.01 | 0.03 | 0.02 | 0.03 | 0.02 | 0.00 | 0.03 | 0.01 | 0.01 |
| BkF | 0.02 | 0.02 | 0.02 | 0.02 | 0.02 | 0.02 | 0.02 | 0.01 | 0.01 |
| BaP | 0.01 | 0.00 | 0.01 | 0.02 | 0.01 | 0.01 | 0.01 | 0.02 | 0.01 |
| DBA | 0.01 | 0.02 | 0.00 | 0.01 | 0.00 | 0.00 | 0.00 | 0.00 | 0.01 |
| IP | 0.02 | 0.05 | 0.00 | 0.03 | 0.04 | 0.01 | 0.02 | 0.01 | 0.02 |
| BghiP | 0.03 | 0.02 | 0.02 | 0.05 | 0.02 | 0.03 | 0.03 | 0.02 | 0.04 |

Table S6. Concentrations of 16 PM_2.5_-associated BaP_eq_ detected during daytime

| Species | Average (ng m^-3^) | | | | | | | | |
| --- | --- | --- | --- | --- | --- | --- | --- | --- | --- |
|  | Sep | Oct | Nov | Dec | Jan | Feb | Mar | Apr | May |
| Nap | 0.00E+00 | 0.00E+00 | 0.00E+00 | 0.00E+00 | 0.00E+00 | 0.00E+00 | 0.00E+00 | 0.00E+00 | 0.00E+00 |
| Aceny | 1.25E-05 | 0.00E+00 | 0.00E+00 | 0.00E+00 | 0.00E+00 | 0.00E+00 | 0.00E+00 | 3.75E-06 | 0.00E+00 |
| Acen | 3.00E-06 | 3.00E-06 | 0.00E+00 | 0.00E+00 | 0.00E+00 | 0.00E+00 | 2.75E-06 | 1.28E-05 | 0.00E+00 |
| Fluo | 4.00E-05 | 2.30E-05 | 0.00E+00 | 1.50E-05 | 2.80E-05 | 0.00E+00 | 6.25E-06 | 3.13E-05 | 6.75E-06 |
| Ph | 1.75E-05 | 1.25E-04 | 6.58E-05 | 1.27E-04 | 1.22E-04 | 1.10E-04 | 1.24E-04 | 2.60E-05 | 0.00E+00 |
| An | 1.18E-04 | 1.78E-04 | 0.00E+00 | 7.25E-05 | 6.25E-05 | 1.25E-04 | 1.13E-04 | 2.18E-04 | 1.43E-04 |
| Flt | 6.03E-05 | 1.33E-04 | 7.43E-05 | 9.73E-05 | 6.08E-05 | 1.37E-04 | 1.24E-04 | 5.40E-05 | 2.73E-05 |
| Py | 4.45E-05 | 1.07E-04 | 5.38E-05 | 6.88E-05 | 4.50E-05 | 9.15E-05 | 9.80E-05 | 6.53E-05 | 2.43E-05 |
| BaA | 2.28E-03 | 5.18E-03 | 2.48E-03 | 3.35E-03 | 2.70E-03 | 4.43E-03 | 3.78E-03 | 2.95E-03 | 1.68E-03 |
| Chry+TriPhe | 7.05E-04 | 1.68E-03 | 6.98E-04 | 9.00E-04 | 7.18E-04 | 1.15E-03 | 1.09E-03 | 8.58E-04 | 3.43E-04 |
| BbF | 5.40E-03 | 1.86E-02 | 8.58E-03 | 1.73E-02 | 1.49E-02 | 1.90E-02 | 1.53E-02 | 6.03E-03 | 3.15E-03 |
| BkF | 6.50E-03 | 1.73E-02 | 1.24E-02 | 1.35E-02 | 8.45E-03 | 1.80E-02 | 8.63E-03 | 5.13E-03 | 2.35E-03 |
| BaP | 3.30E-02 | 9.23E-02 | 5.30E-02 | 1.02E-01 | 4.53E-02 | 1.01E-01 | 6.10E-02 | 3.55E-02 | 1.53E-02 |
| DBA | 3.15E-02 | 4.83E-02 | 0.00E+00 | 3.00E-03 | 1.80E-02 | 8.75E-03 | 1.70E-02 | 1.00E-02 | 2.30E-02 |
| IP | 5.40E-03 | 1.72E-02 | 7.28E-03 | 1.94E-02 | 2.62E-02 | 1.56E-02 | 1.38E-02 | 5.75E-03 | 3.80E-03 |
| BghiP | 1.96E-03 | 3.24E-03 | 2.75E-03 | 3.77E-03 | 2.44E-03 | 3.65E-03 | 2.23E-03 | 1.30E-03 | 1.15E-03 |
|  | Standard deviation (ng m^-3^) | | | | | | | | |
| Nap | 0.00E+00 | 0.00E+00 | 0.00E+00 | 0.00E+00 | 0.00E+00 | 0.00E+00 | 0.00E+00 | 0.00E+00 | 0.00E+00 |
| Aceny | 2.50E-05 | 0.00E+00 | 0.00E+00 | 0.00E+00 | 0.00E+00 | 0.00E+00 | 0.00E+00 | 7.50E-06 | 0.00E+00 |
| Acen | 6.00E-06 | 6.00E-06 | 0.00E+00 | 0.00E+00 | 0.00E+00 | 0.00E+00 | 5.50E-06 | 2.22E-06 | 0.00E+00 |
| Fluo | 1.82E-05 | 1.70E-05 | 0.00E+00 | 3.00E-05 | 2.73E-05 | 0.00E+00 | 1.25E-05 | 7.09E-06 | 1.35E-05 |
| Ph | 3.50E-05 | 2.19E-05 | 4.89E-05 | 1.07E-04 | 5.06E-05 | 2.11E-05 | 3.72E-05 | 3.03E-05 | 0.00E+00 |
| An | 1.03E-04 | 2.99E-05 | 0.00E+00 | 8.85E-05 | 7.32E-05 | 2.08E-05 | 1.70E-04 | 7.18E-05 | 2.22E-05 |
| Flt | 3.59E-05 | 2.05E-05 | 2.39E-05 | 5.44E-05 | 1.63E-05 | 2.09E-05 | 3.64E-05 | 1.28E-05 | 4.50E-06 |
| Py | 2.78E-05 | 1.57E-05 | 1.70E-05 | 3.49E-05 | 1.25E-05 | 1.12E-05 | 3.65E-05 | 8.14E-06 | 3.40E-06 |
| BaA | 1.37E-03 | 1.50E-04 | 9.74E-04 | 8.96E-04 | 5.29E-04 | 5.74E-04 | 9.91E-04 | 4.65E-04 | 2.50E-04 |
| Chry+TriPhe | 4.27E-04 | 1.36E-04 | 1.05E-04 | 2.65E-04 | 1.42E-04 | 1.66E-04 | 3.93E-04 | 2.16E-04 | 5.38E-05 |
| BbF | 3.06E-03 | 3.46E-03 | 1.70E-03 | 3.45E-03 | 5.29E-04 | 1.75E-03 | 2.75E-03 | 4.57E-04 | 5.80E-04 |
| BkF | 3.52E-03 | 2.26E-03 | 1.34E-03 | 3.51E-03 | 1.06E-03 | 3.08E-03 | 1.89E-03 | 4.11E-04 | 5.20E-04 |
| BaP | 2.09E-02 | 1.30E-02 | 1.15E-02 | 2.17E-02 | 4.50E-03 | 1.10E-02 | 1.68E-02 | 6.95E-03 | 1.19E-02 |
| DBA | 1.72E-02 | 1.53E-02 | 0.00E+00 | 6.00E-03 | 4.69E-03 | 6.60E-03 | 4.55E-03 | 1.01E-02 | 6.88E-03 |
| IP | 3.33E-03 | 2.99E-03 | 9.11E-04 | 2.19E-03 | 1.66E-03 | 2.79E-03 | 1.77E-03 | 7.14E-04 | 4.24E-04 |
| BghiP | 1.06E-03 | 3.97E-04 | 3.21E-04 | 5.73E-04 | 9.31E-05 | 3.09E-04 | 1.42E-04 | 1.12E-04 | 1.78E-04 |

Table S7. Concentrations of 16 PM_2.5_-associated BaP_eq_ detected during nighttime

| Species | Average (ng m^-3^) | | | | | | | | |
| --- | --- | --- | --- | --- | --- | --- | --- | --- | --- |
|  | Sep | Oct | Nov | Dec | Jan | Feb | Mar | Apr | May |
| Nap | 0.00E+00 | 0.00E+00 | 0.00E+00 | 0.00E+00 | 0.00E+00 | 0.00E+00 | 0.00E+00 | 0.00E+00 | 0.00E+00 |
| Aceny | 0.00E+00 | 0.00E+00 | 0.00E+00 | 0.00E+00 | 0.00E+00 | 0.00E+00 | 1.28E-05 | 3.75E-06 | 0.00E+00 |
| Acen | 0.00E+00 | 0.00E+00 | 0.00E+00 | 0.00E+00 | 0.00E+00 | 0.00E+00 | 1.00E-05 | 1.00E-05 | 2.50E-06 |
| Fluo | 5.25E-06 | 1.15E-05 | 4.75E-06 | 1.13E-05 | 1.85E-05 | 0.00E+00 | 2.15E-05 | 2.00E-05 | 9.25E-06 |
| Ph | 6.63E-05 | 6.83E-05 | 9.08E-05 | 1.60E-04 | 1.10E-04 | 1.21E-04 | 1.64E-04 | 0.00E+00 | 1.35E-05 |
| An | 4.75E-05 | 8.00E-05 | 4.75E-05 | 4.50E-05 | 1.60E-04 | 8.75E-05 | 2.98E-04 | 2.30E-04 | 1.30E-04 |
| Flt | 3.80E-05 | 4.20E-05 | 7.95E-05 | 1.57E-04 | 6.80E-05 | 9.88E-05 | 1.65E-04 | 3.48E-05 | 2.23E-05 |
| Py | 3.38E-05 | 3.78E-05 | 5.28E-05 | 9.28E-05 | 5.30E-05 | 8.03E-05 | 1.28E-04 | 4.50E-05 | 2.15E-05 |
| BaA | 2.95E-03 | 3.08E-03 | 4.23E-03 | 3.38E-03 | 2.98E-03 | 2.68E-03 | 4.03E-03 | 9.25E-04 | 9.00E-04 |
| Chry+TriPhe | 6.95E-04 | 8.08E-04 | 8.98E-04 | 1.07E-03 | 1.14E-03 | 8.35E-04 | 1.42E-03 | 2.88E-04 | 2.55E-04 |
| BbF | 1.60E-02 | 1.37E-02 | 1.28E-02 | 2.04E-02 | 3.20E-02 | 1.44E-02 | 1.86E-02 | 2.75E-03 | 6.13E-03 |
| BkF | 1.72E-02 | 1.59E-02 | 1.82E-02 | 1.94E-02 | 1.50E-02 | 1.38E-02 | 1.32E-02 | 3.05E-03 | 4.95E-03 |
| BaP | 5.20E-02 | 6.38E-02 | 6.80E-02 | 9.03E-02 | 5.13E-02 | 5.40E-02 | 7.83E-02 | 2.20E-02 | 2.20E-02 |
| DBA | 3.93E-02 | 4.53E-02 | 1.08E-02 | 6.75E-03 | 3.20E-02 | 1.75E-03 | 2.35E-02 | 0.00E+00 | 2.95E-02 |
| IP | 1.71E-02 | 2.49E-02 | 9.58E-03 | 1.95E-02 | 4.77E-02 | 1.17E-02 | 1.71E-02 | 2.95E-03 | 7.93E-03 |
| BghiP | 3.41E-03 | 3.16E-03 | 3.12E-03 | 3.45E-03 | 4.55E-03 | 3.19E-03 | 2.08E-03 | 8.88E-04 | 1.56E-03 |
|  | Standard deviation (ng m^-3^) | | | | | | | | |
| Nap | 0.00E+00 | 0.00E+00 | 0.00E+00 | 0.00E+00 | 0.00E+00 | 0.00E+00 | 0.00E+00 | 0.00E+00 | 0.00E+00 |
| Aceny | 0.00E+00 | 0.00E+00 | 0.00E+00 | 0.00E+00 | 1.56E-05 | 7.50E-06 | 0.00E+00 | 7.36E-06 | 2.17E-06 |
| Acen | 0.00E+00 | 0.00E+00 | 0.00E+00 | 0.00E+00 | 7.66E-06 | 6.65E-06 | 5.00E-06 | 6.43E-06 | 5.77E-06 |
| Fluo | 1.10E-05 | 1.22E-05 | 1.17E-05 | 2.38E-06 | 1.51E-05 | 1.24E-05 | 1.85E-05 | 6.64E-06 | 3.21E-06 |
| Ph | 6.00E-06 | 5.25E-05 | 2.81E-05 | 2.64E-05 | 7.55E-05 | 5.51E-05 | 5.76E-05 | 8.24E-05 | 7.17E-05 |
| An | 5.51E-05 | 3.51E-05 | 6.40E-05 | 5.59E-05 | 1.66E-04 | 4.12E-05 | 3.84E-05 | 1.09E-04 | 4.16E-05 |
| Flt | 6.51E-06 | 6.15E-05 | 1.76E-05 | 2.01E-05 | 8.95E-05 | 1.88E-05 | 3.95E-05 | 6.89E-05 | 5.86E-05 |
| Py | 3.79E-06 | 3.14E-05 | 1.26E-05 | 1.78E-05 | 6.06E-05 | 8.43E-06 | 3.00E-05 | 5.61E-05 | 3.98E-05 |
| BaA | 7.81E-04 | 3.90E-04 | 5.84E-04 | 1.13E-03 | 1.01E-03 | 1.26E-03 | 1.12E-03 | 1.39E-03 | 2.38E-03 |
| Chry+TriPhe | 4.58E-05 | 2.13E-04 | 1.78E-04 | 7.74E-05 | 3.63E-04 | 4.45E-04 | 3.04E-04 | 5.64E-04 | 8.08E-04 |
| BbF | 1.93E-03 | 4.10E-03 | 9.70E-03 | 8.63E-04 | 2.75E-03 | 1.48E-02 | 4.10E-03 | 8.50E-03 | 8.49E-03 |
| BkF | 1.47E-03 | 2.47E-03 | 1.80E-03 | 3.10E-03 | 3.91E-03 | 6.08E-03 | 4.77E-03 | 4.85E-03 | 8.26E-03 |
| BaP | 1.50E-02 | 2.65E-02 | 8.76E-03 | 1.64E-02 | 1.42E-02 | 2.16E-02 | 1.79E-02 | 3.11E-02 | 3.61E-02 |
| DBA | 5.03E-03 | 1.65E-02 | 6.31E-03 | 5.35E-03 | 8.63E-03 | 1.60E-02 | 1.74E-02 | 1.61E-03 | 2.35E-02 |
| IP | 5.57E-04 | 3.16E-03 | 1.09E-02 | 1.40E-03 | 2.03E-03 | 2.25E-02 | 2.58E-03 | 7.66E-03 | 7.07E-03 |
| BghiP | 1.97E-04 | 3.59E-04 | 6.74E-04 | 2.82E-04 | 7.60E-04 | 1.87E-03 | 9.16E-04 | 4.59E-04 | 1.25E-03 |

**Reference**

1 Nisbet, I. C. T. & Lagoy, P. K. TOXIC EQUIVALENCY FACTORS (TEFS) FOR POLYCYCLIC AROMATIC-HYDROCARBONS (PAHS). *Regulatory Toxicology and Pharmacology* **16**, 290-300, doi:10.1016/0273-2300(92)90009-x (1992).
